# Cell death resulted from loss of fumarylacetoacetate hydrolase in *Arabidopsis* is related to phytohormone jasmonate but not salicylic acid

**DOI:** 10.1038/s41598-020-70567-0

**Published:** 2020-08-13

**Authors:** Zhou Zhou, Tiantian Zhi, Chengyun Han, Zhihong Peng, Ruozhong Wang, Jianhua Tong, Qi Zhu, Chunmei Ren

**Affiliations:** 1 Hunan Provincial Key Laboratory of Crop Germplasm Innovation and Utilization, Hunan Agricultural University, Changsha, 410128 China; 2 College of Bioscience and Biotechnology, Hunan Agricultural University, Changsha, 410128 China; 3 College of Life Sciences and Resources and Environment, Yichun University, Yichun, 336000 China; 4 College of Horticulture and Landscape, Hunan Agricultural University, Changsha, 410128 China

**Keywords:** Genetics, Molecular biology, Plant sciences

## Abstract

Fumarylacetoacetate hydrolase (FAH) catalyzes the final step in Tyr degradation pathway essential to animals but not well understood in plants. Previously, we found that mutation of *SSCD1* encoding *Arabidopsis* FAH causes cell death under short day, which uncovered an important role of Tyr degradation pathway in plants. Since phytohormones salicylic acid (SA) and jasmonate (JA) are involved in programmed cell death, in this study, we investigated whether *sscd1* cell death is related to SA and JA, and found that (1) it is accompanied by up-regulation of JA- and SA-inducible genes as well as accumulation of JA but not SA; (2) it is repressed by breakdown of JA signaling but not SA signaling; (3) the up-regulation of reactive oxygen species marker genes in *sscd1* is repressed by breakdown of JA signaling; (4) treatment of wild-type *Arabidopsis* with succinylacetone, an abnormal metabolite caused by loss of FAH, induces expression of JA-inducible genes whereas treatment with JA induces expression of some Tyr degradation genes with dependence of JA signaling. These results demonstrated that cell death resulted from loss of FAH in *Arabidopsis* is related to JA but not SA, and suggested that JA signaling positively regulates *sscd1* cell death by up-regulating Tyr degradation.

## Introduction

Programmed cell death (PCD) is a sequence of genetically regulated events resulting in the elimination of specific cells, tissues, or whole organs^1^, which is required both for normal development and to face stress conditions^2–4^. In plants, one well-characterized example of PCD is hypersensitive response taking place on incompatible plant–pathogen interactions^3^, which leads to cell death and then forms visible lesions at the site infected by an avirulent pathogen, as a result, limits the pathogen spread^4^. Phytohormones including salicylic acid (SA) and jasmonate (JA) appear to be key players for hypersensitive response regulation^5^.

To date, a large number of mutants displaying spontaneous cell death lesions have been identified in plants including *Arabidopsis*, rice, barley, maize, and so on^6–9^. These mutants have been named as lesion-mimic mutants (LMM) because of the form of lesions in the absence of pathogen infection^10^. In some of LMM, the SA or JA signaling has been activated^9,11^. By isolating LMM’s genes, many of regulators that play important roles in PCD and SA or JA signal defense responses have been identified, including ACCELERATED CELL DEATH11, LESION SIMULATING DISEASE1, and NICOTIANA BENTHAMIANA HOMEOBO1^12–14^.

SA is involved in plant defense and cell death^15,16^. The level of SA correlates with the expression of *PATHOGENESIS-RELATED1* (*PR1*) gene and resistance to pathogen attack^17,18^. The *NON-EXPRESSOR OF PATHOGENESIS-RELATED GENES1* (*NPR1*) gene is required for SA-induced expression of *PR1* gene and resistance in *Arabidopsis*^19,20^.

Jasmonates (JAs) including jasmonic acid, methyl jasmonate (MeJA), and other derivatives, are a basic class of plant hormones involved in different processes, including plant growth, development, and responses to biotic and abiotic stresses^21–23^. JA signaling pathway is closely involved in plant PCD^24,25^. The F-box protein CORONATINE INSENSITIVE1 (COI1) has been found to be an indispensable component of the JA signaling pathway^26–28^. JA induces expression of many genes including those for vegetative storage proteins (*VSPs*), a thionin (*THI2.1*), and a plant defensin (*PDF1.2*), which is abolished in the *coi1* mutant^28,29^.

In plants, PCD also correlates to reactive oxygen species (ROS), which produces in plants as byproducts of aerobic metabolism and controls a variety of physiological functions including responses to abiotic and biotic stress and plant growth and development^30,31^. The generation of ROS is one of the most normal responses to PCD^32–34^ and the genes associated with oxidative stress are up-regulated during PCD^35–38^. For example, the expression of *ascorbate peroxidase 2* (*APX2*) is rapidly induced by oxidative stress^37^. Oxidative signal inducible 1 (OXI1) is regulated by ROS and the *OXI1* expression is specifically induced by stress conditions that cause cell death^38,39^. The expressions of *bonzai1-associated protein1* (*BAP1*) and a putative *c2h2 zinc finger transcription factor (ZP)* are induced specifically by singlet oxygen, one form of ROS^40^.

In addition, plant PCD is resulted from blockage of some metabolic pathways such as Tyr degradation^41^, an essential pathway to animals^42^. The Tyr degradation pathway includes five-step enzymatic reactions^42^. First, Tyr aminotransferase (TAT) catalyzes the conversion of Tyr into 4-hydroxyphenylpyruvate, which is then converted into homogentisate by 4-hydroxyphenylpyruvate dioxygenase. Next, homogentisate dioxygenase (HGO) catalyzes homogentisate to yield maleylacetoacetate that is isomerized by maleylacetoacetate isomerase (MAAI) to fumarylacetoacetate, and finally fumarylacetoacetate hydrolase (FAH) hydrolyses fumarylacetoacetate to fumarate and acetoacetate^42^. Loss of FAH results in the accumulation of fumarylacetoacetate and maleylacetoacetate, both of which would undergo spontaneous reduction to succinylacetoacetate that is converted to succinylacetone (SUAC) by spontaneous nonenzymatic decarboxylation^42^. SUAC is toxic to cells and tissues resulting in severe metabolic disorder diseases in mammals^42–44^. In *Arabidopsis*, we have identified one LMM named as *short-day sensitive cell death1* (*sscd1*) displaying spontaneous cell death lesions under short day (SD) conditions, and isolated the *SSCD1* gene encoding the *Arabidopsis* putative FAH, which uncovered the role of Tyr degradation pathway in plant^41^.

To investigate whether the appearance of spontaneous cell death lesions in the *sscd1* mutant is related to SA and JA, in this study, we first analyzed expression of some SA- and JA-inducible genes and then generated double mutants of *sscd1* with *npr1* and *coi1*, respectively, and found that cell death in *sscd1* is accompanied by JA accumulation and repressed by mutation of *COI1*, however, it is unrelated to SA although it is accompanied by up-regulation of SA-inducible *PR1*. Furthermore, we found that the up-regulation of ROS marker genes such as *APX2*, *OXI1*, *BAP1*, and *ZP* in the *sscd1* mutant is also repressed by mutation of *COI1*. In addition, we found that treatment of *Arabidopsis* seedlings with SUAC induces expression of JA-inducible genes. However, treatment with JA induces expression of some Tyr degradation pathway genes including *TAT3* encoding an *Arabidopsis* putative TAT^45^, *HGO*, and *MAAI*, which is dependence of *COI1*. Our work uncovered a crosstalk between JA signaling and Tyr degradation pathway in the regulation of *sscd1* cell death, i.e. JA signaling positively regulates *sscd1* cell death by up-regulating Tyr degradation.

## Results

### Cell death in *sscd1* is uncorrelated to SA signaling although it is accompanied by up-regulation of SA-inducible *PR1*

**Figure 1:**
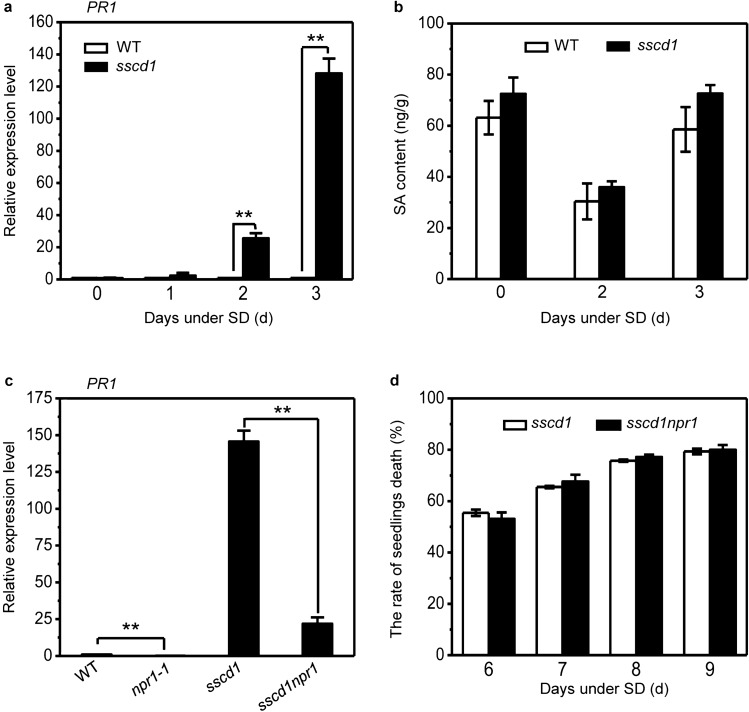
Cell death in *sscd1* is uncorrelated to SA signaling although it is accompanied by the up-regulation of SA-inducible *PR1*. (**a**) Relative expression level of SA-inducible genes *PR1* in wild-type (WT) and *sscd1* seedlings that were grown under LD for 3 weeks, and then transferred to SD for 0, 1, 2 and 3 days. (**b**) The content of SA in wild-type (WT) and *sscd1* seedlings that were grown under LD for 3 weeks, and then transferred to SD for 0, 2 and 3 days. (**c**) Relative expression level of SA-inducible genes *PR1* in wild-type (WT), *npr1-1, sscd1* and *sscd1npr1* seedlings that were grown under LD for 3 weeks, and then transferred to SD for 3 days. (**d**) The rate of seedlings death in *sscd1* and *sscd1npr1* seedlings grown on MS under SD for 6–9 days. LD, long day; SD, short day. The expression of gene was analyzed by RT-qPCR, relative expression level was normalized to those of *ACTIN2* and the control (in wild type) was set to 1. Mean ± SE from three biological replicates. Asterisk ** represents the significance of differences (two-tailed Student’s t-test) at the level of P < 0.01.

The *sscd1* mutant grows normally under long day (LD), but displays obvious cell death symptoms after transferred to SD for 3 days^41^. To investigate whether cell death in *sscd1* is related to SA, we first analyzed expression of *PR1*, one of SA-inducible genes, in wild-type and *sscd1* seedlings transferred from LD to SD for 1, 2 and 3 days by quantitative real-time polymerase chain reaction (RT-qPCR). As shown in Fig. 1a, no significant difference in the expression level of *PR1* between wild type and *sscd1* was observed before seedlings were transferred to SD or after they were transferred to SD for 1 day, however, the expression level of *PR1* was significantly increased in *sscd1* compared to wild type when seedlings were transferred to SD for 2 days and that this increase was much more obvious after seedlings were transferred to SD for 3 days.

Since SA-inducible gene *PR1* was significantly up-regulated in the *sscd1* mutant compared to wild type when seedlings were transferred from LD to SD for 2–3 days (Fig. 1a), we next measured the content of SA to investigate whether up-regulation of *PR1* is resulted from accumulation of SA in the *sscd1* mutant. Unexpected, the content of SA was not significantly increased in *sscd1* compared to wild type before seedlings were transferred to SD or after they were transferred to SD for 2 or 3 days (Fig. 1b). Therefore, the up-regulation of *PR1* in *sscd1* was not related to SA.

NPR1 is a SA receptor in SA signaling^46^ and expression of SA-inducible *PR1* is abolished in the *npr1* mutant^19^. To investigate whether loss of NPR1 influences the up-regulation of *PR1* as well as the cell death in *sscd1*, a double mutant of *sscd1* and *npr1-1* was generated and then expression of *PR1* was analyzed as well as the seedlings phenotype was observed. As shown in Fig. 1c, expression of *PR1* was almost undetected in the *npr1-1* mutant whereas it was significantly induced in the *sscd1npr1* double mutant although its level was much lower than that in the *sscd1* single mutant after seedlings were transferred to SD for 3 days. Furthermore, the rate of seedlings death in *sscd1npr1* was similar to that in *sscd1* (Fig. 1d). These results demonstrated that both up-regulation of *PR1* and cell death in *sscd1* are independent of NPR1 and that cell death in *sscd1* is uncorrelated to SA signaling although it is accompanied by the up-regulation of SA-inducible *PR1*.

### Cell death in *sscd1* is accompanied by up-regulation of JA-inducible genes and accumulation of jasmonic acid

**Figure 2:**
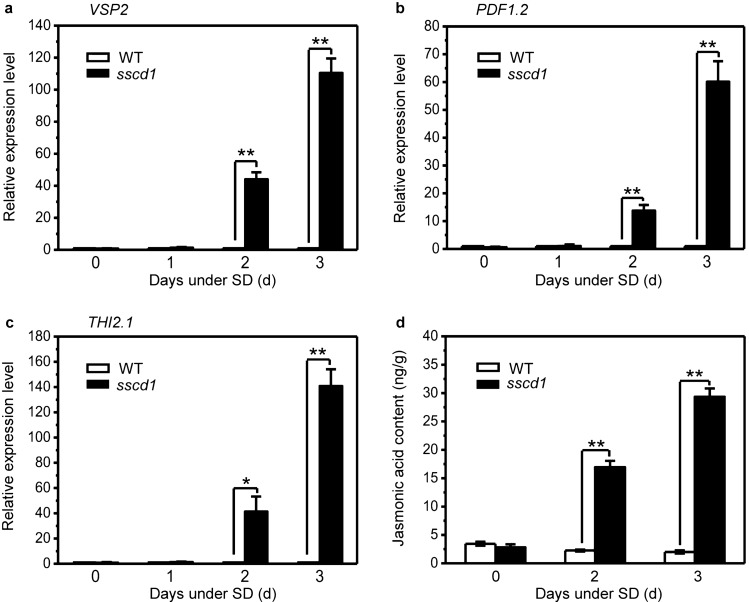
Cell death in *sscd1* is accompanied by the up-regulation of JA-inducible genes and the accumulation of jasmonic acid. (**a**–**c**) Relative expression level of JA -inducible genes *VSP2* (**a**), *PDF1.2* (**b**), *THI2.1* (**c**) in wild-type (WT) and *sscd1* seedlings that were grown under LD for 3 weeks, and then transferred to SD for 0, 1, 2 and 3 days. (**d**) The content of jasmonic acid in wild-type (WT) and *sscd1* seedlings that were grown under LD for 3 weeks, and then transferred to SD for 0, 2 and 3 days. LD, long day; SD, short day. The expression of genes was analyzed by RT-qPCR, relative expression level was normalized to those of *ACTIN2* and the control (in wild type) was set to 1. Mean ± SE from three biological replicates. Asterisk * and ** represent the significance of differences (two-tailed Student’s t-test) at the levels of P < 0.05 and P < 0.01, respectively.

Since cell death in *sscd1* is uncorrelated to SA signaling (Fig. 1), we next investigated whether it is related to JA signaling. We first analyzed the expression of JA-inducible genes including *VSP2*, *PDF1.2*, and *THI2.1* in wild-type and *sscd1* seedlings which were transferred from LD to SD for 1, 2 and 3 days. The results showed that the expression level of these genes was similar in wild type and *sscd1* before seedlings were transferred to SD or after they were transferred to SD for 1 day, however, it was significantly increased in *sscd1* compared to wild type after seedlings were transferred to SD for 2 days and that this increase was much more obvious after seedlings were transferred to SD for 3 days (Fig. 2a–c). Then, we measured the content of jasmonic acid in wild type and *sscd1* before seedlings were transferred to SD or after they were transferred to SD for 2 and 3 days to investigate whether the up-regulation of these genes is resulted from the accumulation of jasmonic acid. The result showed that there was no significant difference in the content of jasmonic acid between wild type and *sscd1* before seedlings were transferred to SD, however, the content of jasmonic acid was significantly increased in *sscd1* compared to that in wild type after seedlings were transferred to SD for 2 days and that this increase was much more distinct after seedlings were transferred to SD for 3 days (Fig. 2d). These results indicated that the cell death in *sscd1* is accompanied by both up-regulation of JA-inducible genes and accumulation of jasmonic acid and suggested that the up-regulation of JA-inducible genes is caused by the accumulation of jasmonic acid.

### Cell death in *sscd1* is repressed by mutation of *COI1*

**Figure 3:**
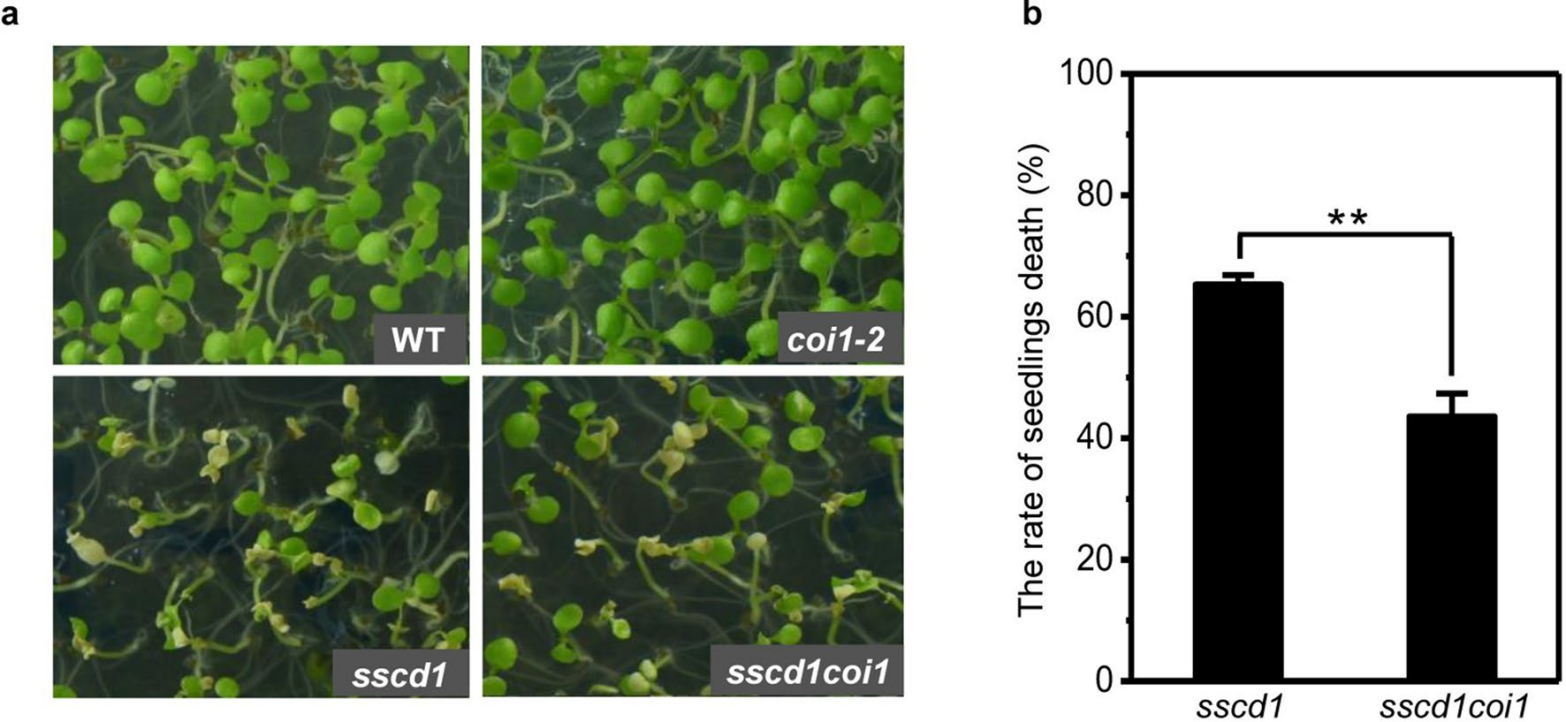
Cell death in *sscd1* is reduced by mutation of *COI1.* (**a**) The phenotype of wild-type (WT), *sscd1*, *coi1-2* and *sscd1coi1* seedlings grown on MS under SD for 7 days. (**b**) The rate of seedlings death in *coi1-2* and *sscd1coi1* seedlings grown on MS under SD for 7 days. SD, short day. Mean ± SE from three biological replicates. Asterisk ** represents the significance of differences (two-tailed student’s t-test) at the level of P < 0.01.

COI1 is a JA receptor in JA signaling^47^. To investigate whether JA signaling mediates the *sscd1* cell death, we generated the *sscd1coi1* double mutant through a cross of *sscd1* with *coi1-2*^28^ to break the JA signaling, and then observed the phenotype of seedlings. It was interesting that the phenotype of seedlings death was obviously rescued in *sscd1coi1* compared to *sscd1* (Fig. 3a). For example, 65% of 7-old *sscd1* seedlings grown under SD were dead whereas the rate of *sscd1coi1* seedlings death was only 43% (Fig. 3b). This result suggested that the cell death in *sscd1* is repressed by breakdown of JA signaling through mutation of *COI1* and that JA signaling positively regulates the *sscd1* cell death.

### Mutation of *COI1* suppresses the up-regulation of ROS marker genes in *sscd1*

**Figure 4:**
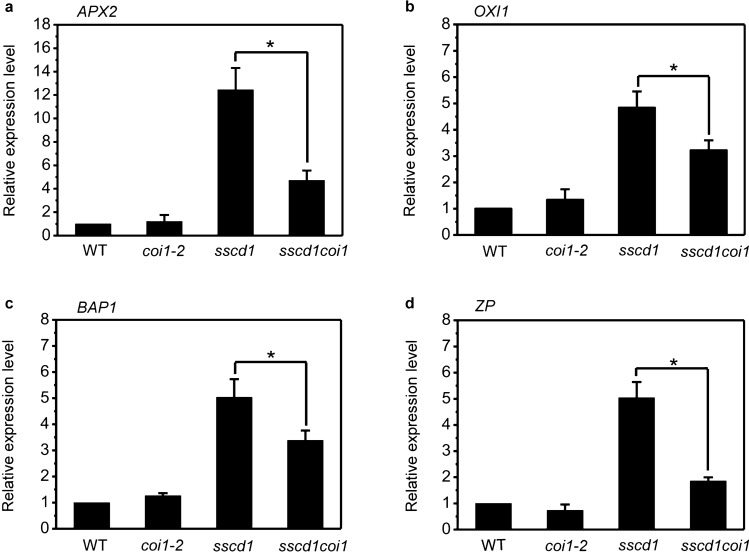
Mutation of *COI1* suppresses the up-regulation of ROS marker genes in *sscd1*. (**a**–**d**) Analysis of the relative expression levels of *APX2* (**a**), *OXI1* (**b**), *BAP1* (**c**) and *ZP* (**d**) in wild-type (WT), *coi1-2*, *sscd1* and *sscd1coi1* seedlings that were grown under SD for 5 days. ROS, Reactive Oxygen Species; SD, short day. The expression of genes was analyzed by RT-qPCR, relative expression level was normalized to those of *ACTIN2* and the control (in wild type) was set to 1. Mean ± SE from three biological replicates. Asterisk * represents the significance of differences (two-tailed Student’s t-test) at the level of P < 0.05.

Previously, we found that ROS marker genes such as *APX2*, *OXI1*, *BAP1* and *ZP* were up-regulated before an occurrence of cell death in the *sscd1* mutant^48^, so, we next investigated whether the repression of cell death in *sscd1* by mutation of *COI1* is correlated with the expression of these genes. Since the cell death phenotype of *sscd1* seedlings that were grown under SD appeared on the 6th day^41,49^, therefore, we tested the expression of *APX2*, *OXI1*, *BAP1* and *ZP* in seedlings grown under SD for 5 days. As shown in Fig. 4, the expression pattern of *APX2*, *OXI1*, *BAP1* and *ZP* was similar in both WT and *coi1-2*, however, the up-regulation of these genes in *sscd1* was significantly suppressed in *sscd1coi1* (Fig. 4), which indicated that the up-regulation of ROS marker genes in *sscd1* could be suppressed by the mutation of *COI1*.

### SUAC treatment activates the expression of JA-inducible genes

**Figure 5:**
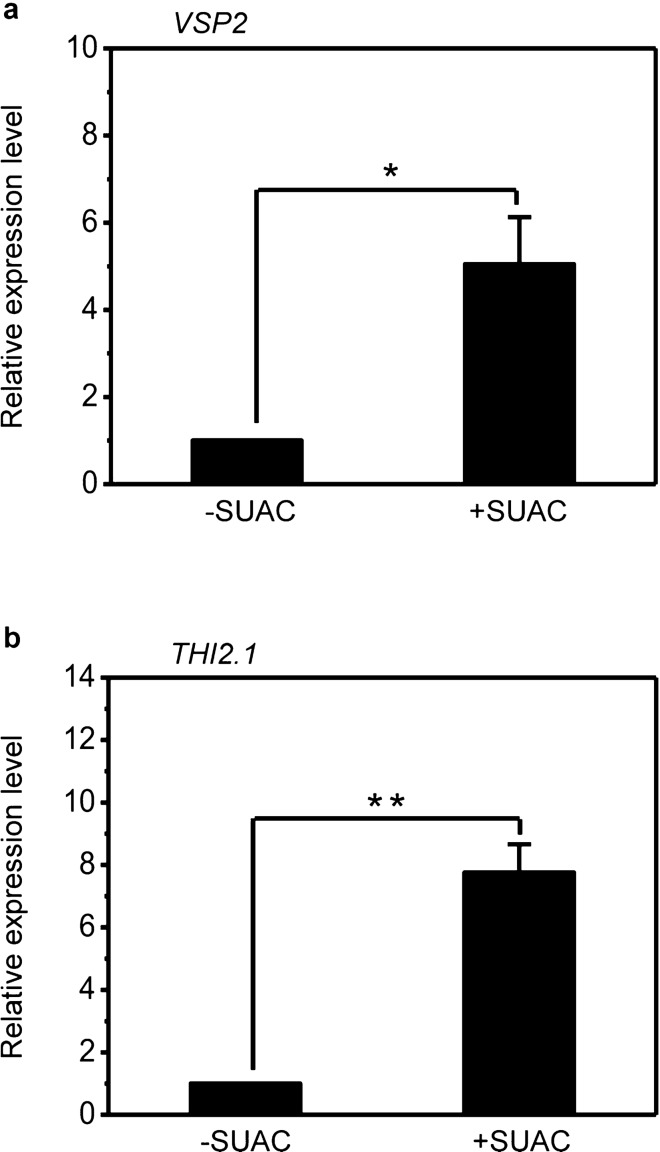
Exogenous SUAC up-regulates the expression of JA-inducible genes *VSP2* and *THI2.1*. (**a**,**b**) Relative expression level of JA-inducible genes *VSP2* (**a**) and *THI2.1* (**b**) in wild-type seedlings that were grown under LD for 3 weeks, and then transferred to SD and treated with ddH_2_0 or 1,280 μg/mL SUAC for 3 days. SUAC, succinylacetone; LD, long day; SD, short day. The expression of genes was analyzed by RT-qPCR, relative expression level was normalized to those of *ACTIN2* and the control (without SUAC treatment) was set to 1. Mean ± SE from three biological replicates. Asterisk * and ** represent the significance of differences (two-tailed Student’s t-test) at the levels of P < 0.05 and P < 0.01, respectively.

Previously, we speculated that the cell death in *sscd1* is resulted from the accumulation of SUAC and also found that treatment of *Arabidopsis* wild-type seedlings with SUAC mimicked the cell death phenotype of *sscd1*^41^. We next investigated whether SUAC treatment activates the expression of JA-inducible genes. To this end, we analyzed the expression of *VSP2* and *THI2.1* in wild-type seedlings treated with SUAC, in which some leaves started wilting. The result showed the expression of both *VSP2* and *THI2.1* was significantly increased upon SUAC treatment (Fig. 5), indicating that SUAC treatment could activate the expression of JA-inducible genes.

### Treatment with MeJA causes the *COI1-*dependent up-regulation of some Tyr degradation pathway genes

**Figure 6:**
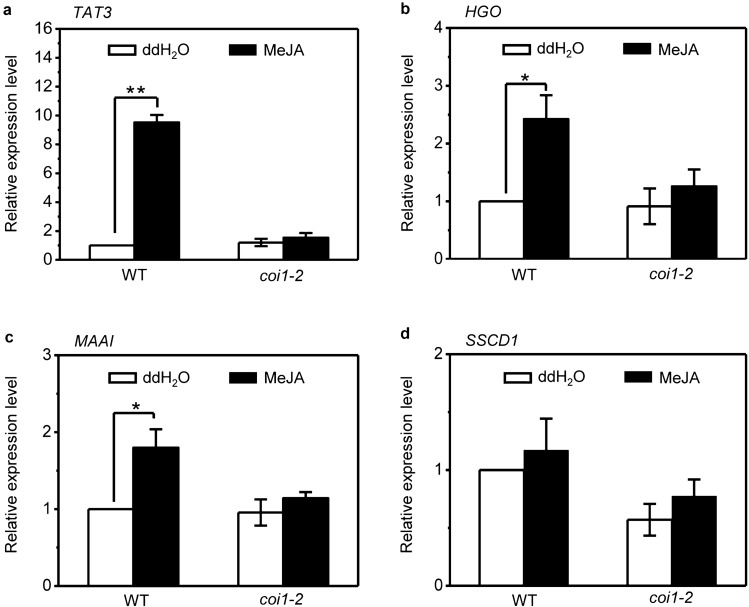
Exogenous MeJA up-regulates the expression of Tyr degradation pathway genes *TAT3*, *HGO*, and *MAAI* and this up-regulation is dependent on *COI1*. (**a**–**d**) Relative expression level of Tyr degradation pathway genes *TAT3* (**a**), *HGO* (**b**), *MAAI* (**c**) and *SSCD1* (**d**) in wild-type (WT) and *coi1-2* seedlings that were grown under LD for 7 days, and then transferred to SD and treated with ddH_2_0 or 100 μM MeJA for 3 days. MeJA, methyl jasmonate; LD, long day; SD, short day. The expression of genes was analyzed by RT-qPCR, relative expression level was normalized to those of *ACTIN2* and the control (in wild type without MeJA treatment) was set to 1. Mean ± SE from three biological replicates. Asterisk * and ** represent the significance of differences (two-tailed Student’s t-test) at the levels of P < 0.05 and P < 0.01, respectively.

Since the cell death in *sscd1* is accompanied by the accumulation of jasmonic acid (Fig. 2d) and could be repressed by breakdown of JA signaling through mutation of *COI1* (Fig. 3), we next investigated whether treatment of *Arabidopsis* wild-type and *coi1-2* seedlings with MeJA influences the Tyr degradation pathway by analyzing the expression of Tyr degradation pathway genes including *TAT3*, *HGO*, *MAAI*, and *SSCD1*. The results showed that the expression level of *TAT3*, *HGO*, and *MAAI* except *SSCD1* was significantly increased in wild type upon MeJA treatment (Fig. 6), especially, an increase of *TAT3* expression level in wild type treated with MeJA was much more significant compared with *HGO* and *MAAI* (Fig. 6a–c). However, it was interesting that the expression level of these genes was not significantly increased in the *coi1-2* mutant upon MeJA treatment (Fig. 6). These results suggested that MeJA up-regulates the expression of some Tyr degradation pathway genes, which would promote Tyr degradation, however, the breakdown of JA signaling through mutation of *COI1* could eliminate an effect of JA on Tyr degradation pathway.

## Discussion

Tyr degradation pathway is essential to animals^42^ but it is not well understood in plants. Previously, we found that mutation of *SSCD1* encoding *Arabidopsis* FAH, an enzyme catalyzing the final step of Tyr degradation pathway, results in spontaneous cell death under SD, which uncovered an important role of Tyr degradation pathway in plants^41^. Afterwards, we found that sugar suppresses cell death caused by disruption of FAH in *Arabidopsis*, indicating that Tyr degradation is regulated by sugar in plants^49^. Recently, we found that cell death resulted from loss of FAH in *sscd1* is related to chlorophyll (Chl) biosynthesis, suggesting a crosstalk between Tyr degradation and Chl biosynthetic pathways in mediating the *sscd1* cell death^48^. Phytohormones such as SA and JA are involved in PCD^13–16,24,25,50^. In this study, the investigation whether cell death resulted from loss of FAH in *Arabidopsis* is related to SA and JA would expand our understandings on the regulation of Tyr degradation pathway in plants.

Through testing expression of SA-inducible *PR1* and content of SA, we found that cell death in *sscd1* was accompanied by the up-regulation of SA-inducible *PR1* (Fig. 1a), however, the content of SA was not significantly altered between in *sscd1* and wild type (Fig. 1b), which indicated that the up-regulation of *PR1* in *sscd1* is independent of SA. Similarly, an increase of *PR1* expression in the *loh1* mutant displaying spontaneous cell death phenotype is also independent of SA^51^. Breakdown of SA signaling by mutation of *NPR1* that encodes a receptor of SA^46^ represses expression of *PR1*^19^. In our study, the expression of *PR1* was also repressed in *sscd1npr1* compared to *sscd1* (Fig. 1c), however, the rate of seedlings death was similar in *sscd1npr1* and *sscd1* (Fig. 1d), suggesting that the cell death in *sscd1* is uncorrelated to both SA signaling and the up-regulation of *PR1*. In addition, we also generated the *sscd1nahG* double mutant by crossing *sscd1* with *nahG* harboring a bacterial gene encoding salicylate hydroxylase that catalyzes the decarboxylation of SA^52,53^ and found that the degree of cell death was similar between *sscd1nahG* and *sscd1* (data not shown), indicating that the degradation of SA would not affect the cell death in *sscd1*, which further confirmed the *sscd1* cell death is not related to SA.

However, cell death in *sscd1* was accompanied by the up-regulation of JA-inducible genes as well as the accumulation of jasmonic acid (Fig. 2). The up-regulation of JA-inducible genes in *sscd1* should be resulted from the accumulation of jasmonic acid, but why the cell death of *sscd1* is accompanied by the accumulation of jasmonic acid? In animals, loss of FAH results in the accumulation of Tyr degradation pathway’s abnormal metabolite SUAC that is toxic to cells and tissues resulting in severe metabolic disorder diseases^42^. In plants, we have found that treatment of *Arabidopsis* wild-type seedlings with SUAC mimicked the *sscd1* cell death phenotype^41^ and demonstrated that the cell death of *sscd1* seedlings correlates with the accumulation of SUAC^54^. Recently, we found that SUAC affects Chl biosynthesis, resulting in the generation of ROS and then inducing cell death^48^. Some researcher’s work has shown that JA could be synthesized in response to singlet oxygen that is one form of ROS^25,55^. Singlet oxygen is very unstable and difficult to detect within a cell^55^, however, some genes were specifically induced by singlet oxygen^40^. Recently, we found that the genes induced specifically by singlet oxygen^40^ were up-regulated in *sscd1*^48^, suggesting that an effect of SUAC on Chl biosynthesis results in the generation of singlet oxygen in the *sscd1* mutant. Furthermore, we found that treatment of *Arabidopsis* wild-type seedlings with SUAC activated the expression of JA-inducible genes (Fig. 5). Taken together, we concluded that cell death in *sscd1* was accompanied by the accumulation of JA (Fig. 2d) is due to the synthesis of JA in response to singlet oxygen.

TAT catalyzes the first step in Tyr degradation pathway^56^. For the first time, Titarenko et al.^57^ reported that *TAT* could be induced by wounding as well as by JA. The gene for the F-box protein COI1 was identified for its irreplaceable role in JA signal transduction^26–28^. Mutations in the *COI1* gene result in plants compromised in all known JA responses: defense against biotic and abiotic stresses, growth inhibition, and fertility^26–28^. Titarenko et al.^57^ reported that wounding induced *TAT* in wild type but not in the *coi1* mutant, suggesting that wound-induced *TAT* is dependent on JA signaling. Brosché and Kangasjärvi^58^ reported that expression of *TAT3* encoding *Arabidopsis* putative TAT^45^ was induced by JA. In this study, we not only confirmed that expression of *TAT3* was induced by JA (Fig. 6a) but also found that expression of some of Tyr degradation pathway’s genes including *HGO* and *MAAI* was also induced by JA (Fig. 6b,c), however, the expression of these genes in the *coi1-2* mutant was not significantly induced by JA (Fig. 6a–c), which suggested that JA signaling up-regulates Tyr degradation in plants.

JA plays an important role in cell death regulation. Singlet oxygen- and JA-mediated cell death in irradiated *flu* plants is likely to be a form of PCD^59^. Inactivation of the EXECUTER1 protein abrogates not only singlet oxygen-mediated cell death of *flu* plants but also accumulation of JA, however, inactivation of JA biosynthesis in the *aos/flu* double mutant does not affect singlet oxygen-mediated cell death^55^, hence, JA does not act as second messengers during singlet oxygen-mediated cell death but forms an integral part of a stress-related signaling cascade activated by singlet oxygen that encompasses several signaling pathways known to be activated by abiotic and biotic stressors^55^. In our study, the cell death of *sscd1* seedlings was repressed by mutation of *COI1* (Fig. 3). Accordingly, the up-regulation of ROS-inducible genes *APX2* and *OXI1*, as well as singlet oxygen specifically induced genes *BAP1* and *ZP* was also repressed by mutation of *COI1* (Fig. 4), suggesting that the breakdown of JA signaling reduces the generation of ROS in the *sscd1* mutant. We have just discussed above that JA signaling up-regulates Tyr degradation. Therefore, the accumulation of JA in *sscd1* would promote cell death by up-regulating Tyr degradation producing more SUAC. However, blockage of JA signaling by mutation of *COI1* breaks the action of JA in Tyr degradation in *sscd1*, resulting in repression of cell death.

Taken all above together, we concluded that cell death resulted from loss of FAH in *Arabidopsis* is related to JA but not SA, and proposed a model for the relationship between JA and Tyr degradation pathway in mediating the *sscd1* cell death. In the *sscd1* mutant, the accumulation of SUAC results in the generation of singlet oxygen, which induces cell death as well as JA synthesis. The accumulation of JA in *sscd1* accelerates Tyr degradation by up-regulating Tyr degradation pathway, producing more SUAC, which promotes cell death. Once JA signaling is broken by mutation of *COI1*, the up-regulation of Tyr degradation by JA in *sscd1* is eliminated, reducing production of SUAC, as a result, the *sscd1* cell death is repressed.

## Methods

### Plant material and growth conditions

The *sscd1* mutant was isolated previously in our laboratory^41^. The *coi1-2* mutant^28^ was kindly provided by Professor Xie (Tsinghua University). The *npr1-1* mutant^19^ was obtained from the Arabidopsis Biological Resource Center (ABRC; Ohio State University, Columbus, OH, USA).

Seeds were surfaced sterilized and plated on Murashige & Skoog (MS) medium in which 1% sucrose was added. Plates were chilled at 4 °C in darkness for 3 days and then transferred to a growth chamber with LD (16 h of light/8 h of dark) or SD (8 h of light/16 h of dark) under 150 μmol photons m^−2^ s^−1^, controlled temperature (22 ± 2 °C).

For RT-qPCR analysis and determination of SA and jasmonic acid in Figs. 1 and 2, the seeds were germinated on MS medium and grown under LD for 1 week and then the seedlings were transplanted to a new MS medium for additional 2 weeks’ growth under LD, and then transferred to SD.

### Construction of double mutants

The *sscd1coi1* double mutant was created by first selecting F_2_ individuals from a cross between *sscd1* and *coi1-2* on plates containing 25 mM MeJA by screening for decreased sensitivity to JA^28^, and then F_3_ lines were selected by sequencing the *SSCD1* gene^41^. The primers for sequencing the *SSCD1* gene are as follows: forward primer is 5′-CCTCGTCCTGCCGTCGCTAT-3′ and reverse primer is 5′-CTTGTGGATGGCCCTGACCT-3′.

The *sscd1npr1* double mutant was created by selecting F_2_ individuals from a cross between *sscd1* and *npr1-1* (a recessive mutation with a single base mutation in *NPR1*^19^) by sequencing *SSCD1* and *NPR1*, respectively. The primers for sequencing the *NPR1* gene are as follows: forward primer is 5′-GTGTGCTCTTCATTTCGCTGTTG-3′ and reverse primer is 5′-ACCCGGTGATGTTCTCTTCGTA-3′.

### RT-qPCR analysis

RT-qPCR analysis were performed as described^48^. Total RNA was isolated using TRIZOL reagent (LIFE TECHNOLOGIES, https://www.thermofisher.com/us/en/home/brands/life-technologies.html). After incubation with DNase I (RNase Free, THERMO FISHER SCIENTIFIC, https://www.thermofisher.com/) at 37 °C for 30 min and then at 65 °C for 10 min to remove genomic DNA, RNA concentrations and purities were measured spectrophotometrically using OD260/OD280 and OD260/OD230 ratios (ND-1000, NanoDrop, THERMO FISHER SCIENTIFIC). Complementary DNA was synthesized from the mixture of oligo-dT primers and random primers using a ReverTraAce qPCR RT kit (perfect real time) according to the manufacturer’s instructions (TOYOBO, https://www.toyobo-global.com/).

RT-qPCR was performed in 96-well blocks using a SYBR qPCR mix (ROCHE, https://lifescience.roche.com/) with a BIO-RAD CFX CONNECT Real-Time PCR detection system (https://www.biorad.com/) following the manufacturer’s instructions. The RT-qPCR amplifications were performed under the following conditions: initial denaturation at 95 °C for 10 min, followed by 40 cycles of 95 °C for 15 s and 60 °C for 60 s. The primers of genes tested by RT-qPCR are listed in Table 1, and *ACTIN2* was used as an internal control. The gene expression for each sample was calculated on three analytical replicates, and the relative expression was quantified using the 2^−ΔΔCt^ method. The experiment was performed in three independent biological repeats. The significance of differences between datasets was evaluated using the two-tailed Student’ t-test.

**Table 1:** Primers of genes tested by RT-qPCR.

Gene	Forward primer	Reverse primer
*PR1* (AT2G14610)	5′-AACTACAACTACGCTGCGAACA-3′	5′-CGAGTCTCACTGACTTTCTCCAA-3′
*VSP2* (AT5G24770)	5′-GGATTGAACCCATCATACTCAG-3′	5′-CACGAGACTCTTCCTCACCTTT-3′
*PDF1.2* (AT5G44420)	5′-GCTTCCATCATCACCCTTATC-3′	5′-TTGGCTTCTCGCACAACTT-3′
*THI2.1* (AT1G72260)	5′-GGTTGGGTAAACGCCATTCT-3′	5′-CATTGTTCCGACGCTCCATT-3′
*APX2* (AT3G09640)	5′-ACAAAGTTGAGCCACCTCCT-3′	5′-AAGGTGTGTCCACCAGACAA-3′
*OXI1* (AT3G25250)	5′-GTTGAGGAAATCAAGGGTCATG-3′	5′-TGGACGATATTCTCCACATCC-3′
*BAP1* (AT3G61190)	5′-ATCGGATCCCACCAGAGATTACGG-3′	5′-AATCTCGGCCTCCACAAACCAG-3′
*ZP* (AT5G04340)	5′-TACGAAGGAAAGAACGGAGGC-3′	5′-GGTATCGGCGGTATGTTGAGG-3′
*TAT3* (AT2G24850)	5′-CTCCGCCCATTCCAACTTCA-3′	5′-ATTCAGCCACCGCCCTTCTA-3′
*HGO* (AT5G54080)	5′-GGAGATTGATTTCGTTGATGGGTT-3′	5′-GCGGAGTCTTTCATTCCTGTGTTA-3′
*MAAI* (AT2G02390)	5′-GCTGGACTCTGCTACTGCGA-3′	5′-AGGGCGATACGGACACGATG-3′
*SSCD1* (AT1G12050)	5′-GACTCGCACTTCCCTATCCAG-3′	5′-GACCATCGAAAAGCCCAGCT-3′
*ACTIN2* (AT3G18780)	5′-AGCACTTGCACCAAGCAGCATG-3′	5′-ACGATTCCTGGACCTGCCTCATC-3′

### Determination of the dead seedlings

Seedlings of *sscd1* and *sscd1npr1* were grown under SD and the number of dead seedling (all leaves were completely bleached) was counted from day 6 to 9. Seedlings of *sscd1* and *sscd1coi1* were grown under SD for 7 days and the number of dead seedlings was counted. The rate of seedling death was calculated as the percentage of dead seedlings from 250 to 300 seedlings. At least three independent biological repeats were performed.

### Detection of jasmonic acid and SA

0.5 g of leaves from WT and *sscd1* seedlings that were grown under LD for 3 weeks and then transferred to SD for 0, 2 and 3 days was harvested for jasmonic acid and SA extraction. The harvested tissues were immediately ground to a fine powder in liquid N_2_, and then exposed to extraction buffer (1.0 mL of 80% methanol) at 4 °C overnight. The samples were centrifuged at 10,000*g* for 5 min, and the residues were re-extracted with 0.6 mL of 80% methanol (HPLC grade methanol, Merck, Germany). The supernatants were vacuum freeze dried to dryness at − 60 °C, then dissolved in 200 μL of 0.1 M sodium phosphate buffer (pH 7.8), and extracted with 200 μL of petroleum ether. The aqueous phase was purified using a Waters Sep-Pak C18 cartridge (Waters, USA). The cartridge was washed with 200 μL of ddH_2_O and then eluted with 1.5 mL of 80% methanol. The eluate with 80% methanol was vacuum freeze dried. The dried extract was dissolved in 40 μL of 50% methanol and used for LC/MS assay in a WATERS ACQUITY SQD (LC/MS) system according to Liu et al.^60^.

### MeJA treatments

For MeJA treatment, the seedlings of WT and *coi1-2* were first grown under LD for 7 days, and then transferred to SD for 3 days. Once transferred to SD, plants were sprayed with 100 μM MeJA or ddH_2_O (as a control) under light once per day for 3 days. After treatment for 3 days, the plants were harvested and used for RT-qPCR analysis. The experiment was performed in three independent biological repeats.

### Treatment with SUAC

The seeds were germinated on MS medium and grown under LD for 1 week and then the seedlings were transplanted to a new MS medium for additional 2 weeks’ growth under LD, and then transferred to SD and sprayed with 1,280 μg mL^−1^ SUAC (SIGMA) or ddH_2_O (as a control) twice per day for 3 days. After treatment for 3 days, the plants were harvested and used for RT-qPCR analysis. The concentration of SUAC treatment was determined following our previous work^49^. The experiment was performed in three independent biological repeats.
